# Glutamate tone in preBötzinger complex rhythmogenesis: ambient extracellular glutamate as a candidate excitability bias

**DOI:** 10.1007/s00424-026-03223-7

**Published:** 2026-10-17

**Authors:** Enrique Vázquez-Mendoza, Carol Alejandra Olmos-Pastoresa, Donají Chí-Castañeda, María Leonor López-Meraz, Luis Beltran-Parrazal, Consuelo Morgado-Valle

**Affiliations:** 1 Doctorado en Investigaciones Cerebrales, Universidad Veracruzana, Dr. Castelazo Ayala s/n, Xalapa, 91190 Veracruz Mexico; 2 Instituto de Investigaciones Cerebrales, Universidad Veracruzana, Dr. Castelazo Ayala s/n, Xalapa, 91190 Veracruz Mexico

**Keywords:** Respiratory rhythm generation, preBötzinger complex, glutamate tone, ambient extracellular glutamate, NMDA receptors, metabotropic glutamate receptors

## Abstract

The preBötzinger complex (preBötC) is essential for inspiratory rhythm generation, yet the biological processes that repeatedly establish the excitability state required for neuronal recruitment remain incompletely understood. This unresolved issue is reflected in computational models of respiratory rhythmogenesis, which rely on a phenomenological baseline excitability parameter, $g_{\mathrm {tonic-e}}$, without a defined biological substrate. Ambient extracellular glutamate signaling is well established in other brain regions, but its relevance to the respiratory network remains largely untested. Here, we evaluate the hypothesis that ambient extracellular glutamate outside synaptic clefts–termed glutamate tone–represents one biologically plausible candidate for a distributed extracellular network-state variable in the preBötC. We define glutamate tone as a continuously maintained but dynamically regulated extracellular glutamate signal arising from the balance of glutamate release, diffusion, transporter-mediated clearance, and astrocyte-dependent metabolism. We critically review evidence that extracellular glutamate handling can influence respiratory network activity, while emphasizing that direct evidence for a compartment-specific, rhythmogenesis-relevant glutamate tone remains lacking. We then evaluate candidate glutamate-sensing mechanisms, including peri-/extrasynaptic NMDA receptors and metabotropic glutamate receptors, and place them within the established framework of recurrent excitation, intrinsic membrane conductances, and inhibitory circuitry. We propose that glutamate tone, if present, would not generate the inspiratory burst directly, but could bias the excitability landscape from which neuronal recruitment and synchronization emerge. Finally, we outline experimental criteria for distinguishing a causal excitability bias from glutamate signals that merely reflect ongoing network activity.

## Introduction

The preBötzinger complex (preBötC), located in the ventrolateral medulla, is the core rhythm-generating microcircuit for mammalian inspiration [1]. Recurrent glutamatergic excitation, intrinsic membrane conductances–including persistent sodium current ($I_{NaP}$) and calcium-activated nonspecific cation current ($I_{CAN}$)–and inhibitory circuitry shape neuronal recruitment, burst amplification, synchronization, and termination [2–5]. Less clearly defined are the biological variables that set the interburst excitability state and recruitment probability from which each inspiratory burst repeatedly emerges.

In classical computational models, this unresolved component is commonly represented by $g_{\mathrm {tonic-e}}$, a tonic excitatory conductance required to reproduce rhythmic bursting and state transitions [6]. Although phenomenologically useful, $g_{\mathrm {tonic-e}}$ is biologically composite: it reproduces baseline excitability without specifying the relative contributions of intrinsic currents, leak conductances, neuromodulatory tone, extracellular ionic conditions, direct synaptic excitatory currents, or extracellular transmitter signaling. Thus, the question addressed here is not whether respiratory rhythm is an emergent network property, but whether a measurable extracellular process contributes to the baseline readiness of the preBötC between successive inspiratory bursts.

Ambient extracellular glutamate signaling is well established in other brain regions, where glutamate acting outside the synaptic cleft can produce sustained receptor activation distinct from fast synaptic transmission [7]. Its relevance to the respiratory network, however, remains largely untested. Here, we use *glutamate tone* to refer specifically to ambient extracellular glutamate outside synaptic clefts. This usage is narrower than *glutamatergic tone*, a broader term that can refer more broadly to overall glutamatergic transmission, pathway activity, glutamate receptor activation, or bulk tissue/neurochemical measures of glutamate availability. In the present review, glutamate tone denotes an extracellular signal shaped by glutamate release, diffusion, transporter-mediated clearance, and astrocyte-dependent metabolism, and potentially detected by peri- or extrasynaptic receptor mechanisms.

Whether such a signal operates within the preBötC, and whether it does so at concentrations, locations, and timescales relevant to respiratory rhythmogenesis, remains unknown. We do not propose glutamate tone as an established rhythmogenic mechanism, nor as a replacement for recurrent excitation, intrinsic conductances, or inhibitory microcircuitry. Rather, we evaluate ambient extracellular glutamate as a biologically plausible and experimentally tractable candidate excitability bias in the preBötC. Specifically, we ask whether a glutamate-tone signal can be detected in rhythmogenic compartments, regulated over relevant timescales, sensed by appropriately positioned glutamate receptors, and shown experimentally to alter recruitment probability, synchronization, or rhythm generation in a manner not reducible to phasic synaptic drive.

## Glutamate tone: biological basis and current uncertainties

Glutamate acting outside the synaptic cleft can constitute a signaling modality distinct from fast synaptic transmission, with different spatial reach, kinetics, and receptor targets [8, 9]. However, this principle cannot be assumed to operate in the preBötC, where the relevant concentrations, compartments, receptor targets, and timescales remain unknown.

Glutamate tone is not conceived here as a single, uniformly distributed extracellular pool. It is better understood as compartmental ambient extracellular glutamate shaped by diffusion barriers, transporter density, synaptic activity, glial proximity, and receptor localization [10, 11]. This distinction is essential because peri- or extrasynaptic receptors may sense glutamate concentrations that differ substantially from those estimated by microdialysis, bulk extracellular sampling, or whole-tissue neurochemical measurements.

The relevant concentration range remains debated. Microdialysis and related approaches often report extracellular glutamate in the low-micromolar range, whereas electrophysiological and compartment-based estimates in slices suggest much lower, often nanomolar, concentrations [9, 11]. This discrepancy matters because micromolar glutamate could support broader activation of high-affinity receptors, whereas nanomolar concentrations would imply receptor activation restricted to local microdomains generated by synaptic spillover, locally reduced uptake, or non-synaptic release. Concentration alone is also insufficient to predict receptor activation, since the functional consequence of any extracellular glutamate signal depends on receptor subtype and localization, co-agonist availability, Mg$^{2+}$ block, and desensitization state [8, 9].

**Fig. 1:**
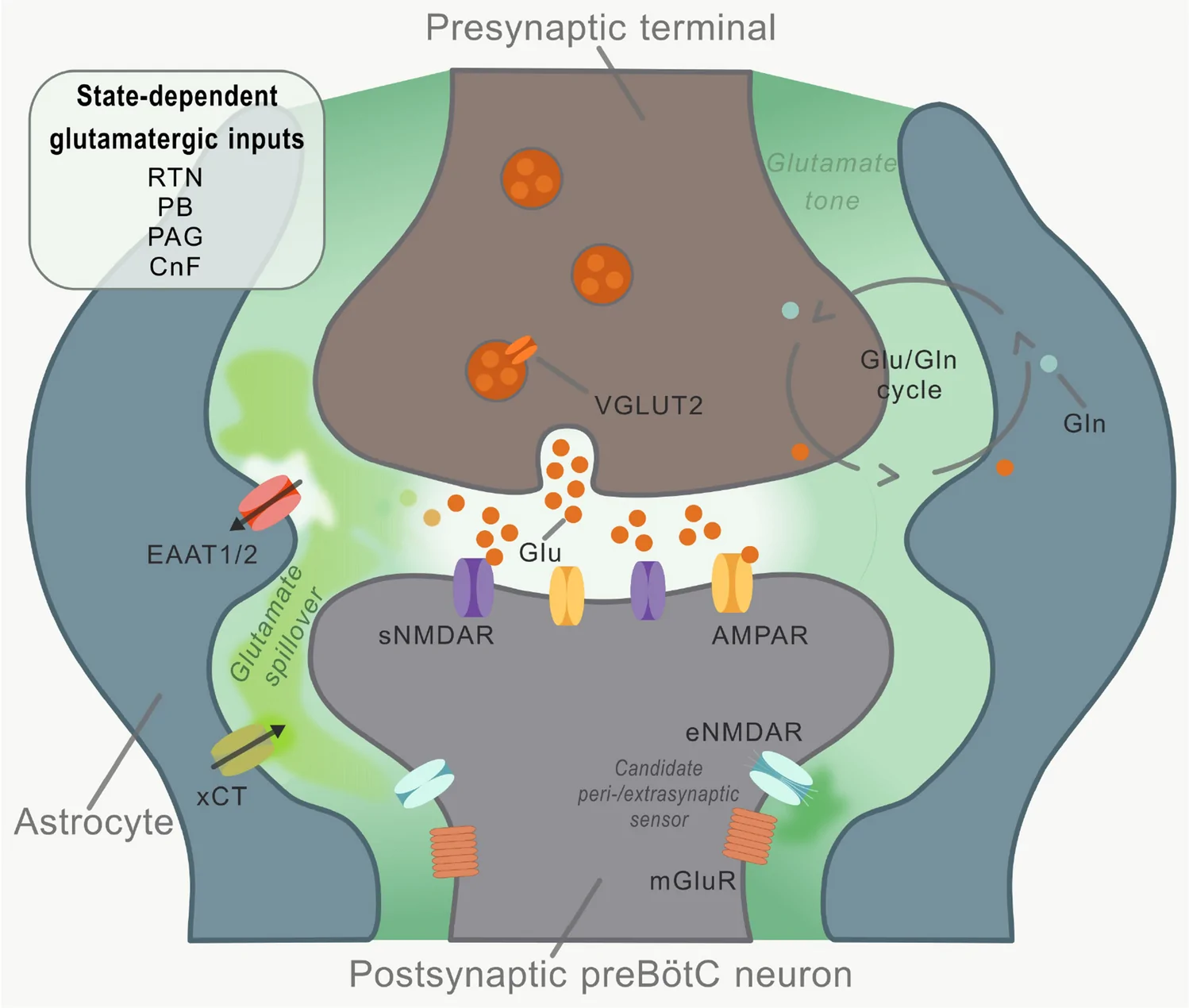
Candidate sources, clearance pathways, and receptor targets of glutamate tone in the preBötC microenvironment. Glutamate tone is proposed to reflect the balance among synaptic spillover from VGLUT2-dependent glutamatergic transmission, sustained afferent glutamatergic input, possible non-synaptic glutamate sources such as xCT, diffusion, astrocytic EAAT1/2-mediated uptake, and astrocyte-neuron glutamate recycling. AMPARs mediate phasic synaptic drive during inspiratory recruitment and bursting, whereas peri-/extrasynaptic NMDARs and mGluRs are candidate mechanisms through which ambient extracellular glutamate may influence neuronal excitability or transmitter release. Extrinsic glutamatergic inputs from respiratory and state-regulatory regions may recalibrate this local balance. Abbreviations: **AMPAR**, AMPA receptor; **CnF**, cuneiform nucleus; **EAAT1/2**, excitatory amino acid transporters 1 and 2; **eNMDAR**, peri- or extrasynaptic NMDA receptor; **Gln**, glutamine; **Glu**, glutamate; **mGluR**, metabotropic glutamate receptor; **PAG**, periaqueductal gray; **PB**, parabrachial nucleus; **RTN**, retrotrapezoid nucleus; **sNMDAR**, synaptic NMDA receptor; **VGLUT2**, vesicular glutamate transporter 2; **xCT**, cystine–glutamate exchanger

If glutamate tone is to have physiological relevance, it must emerge from regulated glutamate homeostasis rather than be postulated as an unspecified background signal. At any location, extracellular glutamate reflects the balance among glutamate release—including synaptic spillover from local recurrent excitation, sustained afferent glutamatergic input, and possible non-synaptic pathways such as the cystine–glutamate exchanger xCT—diffusion, excitatory amino acid transporters (EAAT)-mediated clearance, and astrocyte-dependent glutamate–glutamine metabolism (Fig. 1) [9–11]. Because uptake, diffusion, and metabolic cycling operate with different spatial constraints and time constants, their net balance could generate local extracellular glutamate signals that persist beyond phasic release events without requiring an additional source of excitation.

On this basis, we define glutamate tone as a continuously maintained but dynamically regulated ambient extracellular glutamate signal outside synaptic clefts. “Tone” does not imply a fixed or invariant concentration. It indicates that basal extracellular glutamate is not reduced to zero by uptake and that local concentrations may vary as the balance among release, diffusion, clearance, and metabolism shifts across microdomains, respiratory-cycle phases, or physiological states. Under this definition, glutamate tone is a candidate distributed network-state variable: a signal that could influence excitability beyond individual synapses if appropriate receptor mechanisms are present.

This operational definition sets the criteria for the following sections. To be relevant to preBötC rhythmogenesis, glutamate tone must be detectable in rhythmogenic compartments, dynamically regulated on relevant timescales, sensed by receptors capable of influencing subthreshold excitability, and experimentally distinguishable from direct synaptic receptor activation.

## Evidence and limitations for glutamate tone in the preBötC

The preceding section defined glutamate tone as a candidate ambient extracellular signal arising from the balance among release, diffusion, uptake, and metabolism. The question here is whether preBötC-specific evidence supports the possibility that extracellular glutamate handling is dynamically linked to respiratory network activity. VGLUT2-dependent transmission is required for central respiratory rhythm generation [12], establishing glutamate as the obligatory excitatory transmitter, but not demonstrating that ambient extracellular glutamate outside synaptic clefts contributes to rhythmogenesis. The narrower question is whether glutamate handling can influence preBötC excitability in a manner not reducible to direct synaptic receptor activation.

Several observations indicate that glutamate clearance and astrocyte-dependent handling can constrain respiratory network output. Respiratory-network astrocytes display functional glutamate transport properties, including EAAT2-mediated uptake mechanisms [13]. Inhibition of EAAT-mediated uptake in neonatal medullary slices reversibly increases hypoglossal motor discharge frequency and amplitude [14], supporting the idea that transporter activity normally limits extracellular glutamate availability. However, uptake blockade cannot identify the relevant compartment, because it elevates glutamate at synaptic, perisynaptic, and extrasynaptic sites and may also alter transporter currents and ionic homeostasis. These experiments were performed in neonatal slices maintained in elevated extracellular K$^{+}$ and continuous perfusion, conditions that may alter extracellular glutamate homeostasis; under some ionic conditions, EAATs can mediate glutamate release rather than uptake [15]. Similarly, pharmacological blockade of the Na$^{+}$/K$^{+}$-ATPase disrupts preBötC network activity [16]; because EAAT function depends on Na$^{+}$ gradients, this observation is relevant to glutamate clearance, but is not direct evidence that altered extracellular glutamate mediates the network effect.

Direct measurements provide complementary but limited evidence. Microdialysis in the medullary region containing ventral respiratory group neurons detected extracellular glutamate in the micromolar range [17], a concentration compatible with sustained activation of high-affinity receptors. However, microdialysis averages glutamate over long sampling periods, cannot distinguish synaptic release from ambient extracellular or non-synaptic glutamate, and lacks the spatial resolution required to define preBötC microdomains. Optical glutamate sensors provide better temporal resolution. In preBötC-containing organotypic slice cultures, glutamate-associated fluorescence signals occur around inspiratory bursts [18]. These signals show that extracellular glutamate varies with respiratory network activity, but not that it precedes recruitment or reflects a sustained component rather than burst-associated synaptic release.

Perturbation studies further illustrate the difficulty of isolating glutamate tone. Depletion of substance P- and glutamate-containing afferent terminals with capsaicin abolishes respiratory rhythm in neonatal medullary slices [19], but would reduce both ambient extracellular glutamate and conventional synaptic transmission. Similarly, disruption of glia-neuron metabolic coupling, including glutamine synthetase-dependent glutamate-glutamine cycling, depresses respiratory activity [20], without determining whether the deficit reflects altered glutamate tone, impaired synaptic glutamate resupply, or broader metabolic compromise.

Astrocytic data are also suggestive but not conclusive. Some putative preBötC astrocytes show intracellular Ca$^{2+}$ rises before inspiratory neuronal firing [21], placing astrocytic signaling within the pre-burst interval in which an excitability bias would need to operate. However, this observation does not identify glutamate as the relevant signal. Indeed, a direct study of astrocyte-neuron coupling in the preBötC found that astrocytic Ca$^{2+}$ signaling is not coupled to glutamate release or uptake [22], arguing against assuming that pre-burst astrocytic activity reflects glutamate-mediated modulation.  

Taken together, the available evidence supports a cautious conclusion. Respiratory network output is sensitive to transporter activity, ionic gradients, afferent glutamatergic input, and astrocyte-dependent metabolism; extracellular glutamate can be detected in respiratory regions; and optical sensor signals vary with inspiratory activity. However, none of these findings demonstrates a sustained or slowly varying, compartment-specific glutamate signal that precedes pre-burst recruitment, nor do they establish a causal role for glutamate tone in recruitment timing, burst probability, or synchronization. The principal unresolved issue is whether receptor-mediated effects of ambient extracellular glutamate can be separated experimentally from direct synaptic receptor activation. Genetically encoded glutamate indicators such as iGluSnFR provide a route to test this criterion directly, provided their signals are interpreted with appropriate constraints on sensor affinity, membrane localization, glutamate buffering, diffusion, and fluorescence calibration [23].

## Candidate receptor mechanisms for detecting glutamate tone

AMPA receptor (AMPAR)-mediated recurrent excitation is the major component of the phasic synaptic drive required for inspiratory burst formation and transmission to motor output [14, 24]. This established role is not in question here. The relevant issue is whether ambient extracellular glutamate outside the synaptic cleft could be detected by receptor mechanisms capable of biasing subthreshold excitability before, or during, the transition to neuronal recruitment.

A receptor mechanism capable of transducing glutamate tone would need to be accessible to glutamate outside the synaptic cleft, respond to low extracellular glutamate concentrations, influence excitability or transmitter release over timescales longer than individual synaptic events, and retain functional relevance during the interburst interval, when many preBötC neurons are below threshold. No receptor class can be assumed *a priori* to meet these criteria in the preBötC.

Peri- or extrasynaptic NMDARs (eNMDARs) represent one plausible candidate, but their candidacy is constrained by important limitations. In other brain regions, ambient extracellular glutamate can produce sustained NMDAR activation [8]. Within the preBötC, NMDAR-mediated current can sustain rhythmic activity when AMPAR-mediated transmission is reduced and Mg$^{2+}$ block is relieved [24]. This demonstrates that NMDARs can support respiratory network excitability under permissive experimental conditions. It does not demonstrate that eNMDARs are tonically active during the normal interburst interval or that they detect endogenous glutamate tone under physiological conditions.

Anatomical and molecular features make this possibility plausible but not proven. Dbx1-derived preBötC neurons are largely aspiny, with excitatory synapses formed directly on dendritic shafts [25]. This organization may reduce geometric barriers to glutamate diffusion beyond the synaptic cleft, but it does not demonstrate spillover or extrasynaptic receptor activation. Consistent with this anatomical possibility, recent confocal evidence from the adult rat preBötC shows abundant NR1-positive puncta with limited PSD95 colocalization [26]. This finding is compatible with peri- or extrasynaptic NMDAR localization, but it cannot establish membrane compartmentalization because confocal microscopy does not resolve synaptic, perisynaptic, extrasynaptic, intracellular, or non-neuronal pools.

The main biophysical challenge is Mg$^{2+}$ block. During the interburst interval, the preBötC neurons are not necessarily hyperpolarized; rather, many operate at subthreshold membrane potentials that may gradually approach recruitment threshold. Under these conditions, conventional NMDARs may still be substantially constrained by voltage-dependent Mg$^{2+}$ block, limiting their capacity to generate a tonic depolarizing influence unless local depolarization, receptor subunit composition, co-agonist availability, or receptor localization favors activation. This limitation depends in part on GluN2 subunit composition. Available studies in respiratory-related neurons support expression of multiple GluN2 subunits, including GluN2C and GluN2D, which are relevant to Mg$^{2+}$ sensitivity and receptor kinetics [27]. However, current evidence does not establish the localization, functional properties, or contribution of these receptors to rhythmogenic excitability *in situ*.

The functional literature imposes an important boundary condition on the eNMDAR component of the hypothesis. Rhythmic respiratory-related activity can still be detected in neonatal medullary slices from mice lacking the obligatory NR1 subunit, and acute pharmacological blockade of NMDARs does not abolish rhythm generation under standard neonatal *in vitro* conditions [14, 24, 28]. These findings argue against NMDAR signaling as a universally required substrate for rhythm expression in the same sense as AMPAR-mediated recurrent excitation. They do not, however, exclude the possibility that specific peri- or extrasynaptic NMDAR populations modulate recruitment probability, burst timing, synchronization, or rhythm robustness. Consequently, the available evidence constrains, but does not falsify, the present hypothesis.

Metabotropic glutamate receptors (mGluRs) provide a second class of candidate glutamate sensors and should not be treated as peripheral to the hypothesis. In other systems, group II/III mGluRs are often located at extrasynaptic, frequently presynaptic, sites, where they can sense ambient glutamate and regulate transmitter release [29]. Group I mGluRs, including mGluR5, can show perisynaptic organization compatible with detection of glutamate spillover [30]. In the preBötC, antagonism of mGluR1 or mGluR5 reduces the inspiratory drive potential [31], supporting a role for mGluR-mediated signaling in respiratory network excitability. These data justify including mGluRs in the glutamate-tone framework, but they do not establish that mGluRs detect glutamate tone or bias recruitment probability.

Thus, the receptor problem should not be framed as eNMDARs versus mGluRs. Both are plausible, non-mutually exclusive mechanisms through which ambient extracellular glutamate could influence the preBötC, and either could exert excitatory, inhibitory, or presynaptic effects depending on receptor subtype, localization, and cellular target. The central unresolved issue is anatomical and functional specificity: whether candidate receptors are positioned in the relevant compartments, engaged by endogenous extracellular glutamate during the interburst interval, and capable of altering recruitment timing, burst probability, or synchronization in a manner not reducible to direct synaptic receptor activation.

## A conceptual framework for glutamate tone in the preBötC

The preceding sections support a cautious framework: extracellular glutamate handling can influence respiratory network output, and peri-/extrasynaptic or presynaptic glutamate receptors could, in principle, detect ambient extracellular glutamate. However, available evidence does not demonstrate a compartment-specific, rhythmogenesis-relevant glutamate tone. We therefore consider glutamate tone as a candidate extracellular variable that may contribute to the excitability state from which preBötC recruitment emerges.

Within this framework, ambient extracellular glutamate could reflect both local and afferent influences (Fig. 1). The local component would arise from the balance among glutamate release, diffusion, EAAT-mediated clearance, and astrocyte-dependent metabolism within the preBötC. The afferent component would reflect glutamatergic inputs from respiratory and state-regulatory regions, including the retrotrapezoid nucleus/parafacial region, parabrachial nucleus, cuneiform nucleus, and periaqueductal gray. These influences need not act independently. Sustained or state-dependent afferent activity may contribute to glutamate tone if released glutamate escapes the synaptic cleft, accumulates locally, or is incompletely cleared. Thus, the key distinction is not the source of glutamate, but whether its functional effect is mediated by direct synaptic receptor activation or by ambient extracellular glutamate acting outside synaptic clefts.

**Fig. 2:**
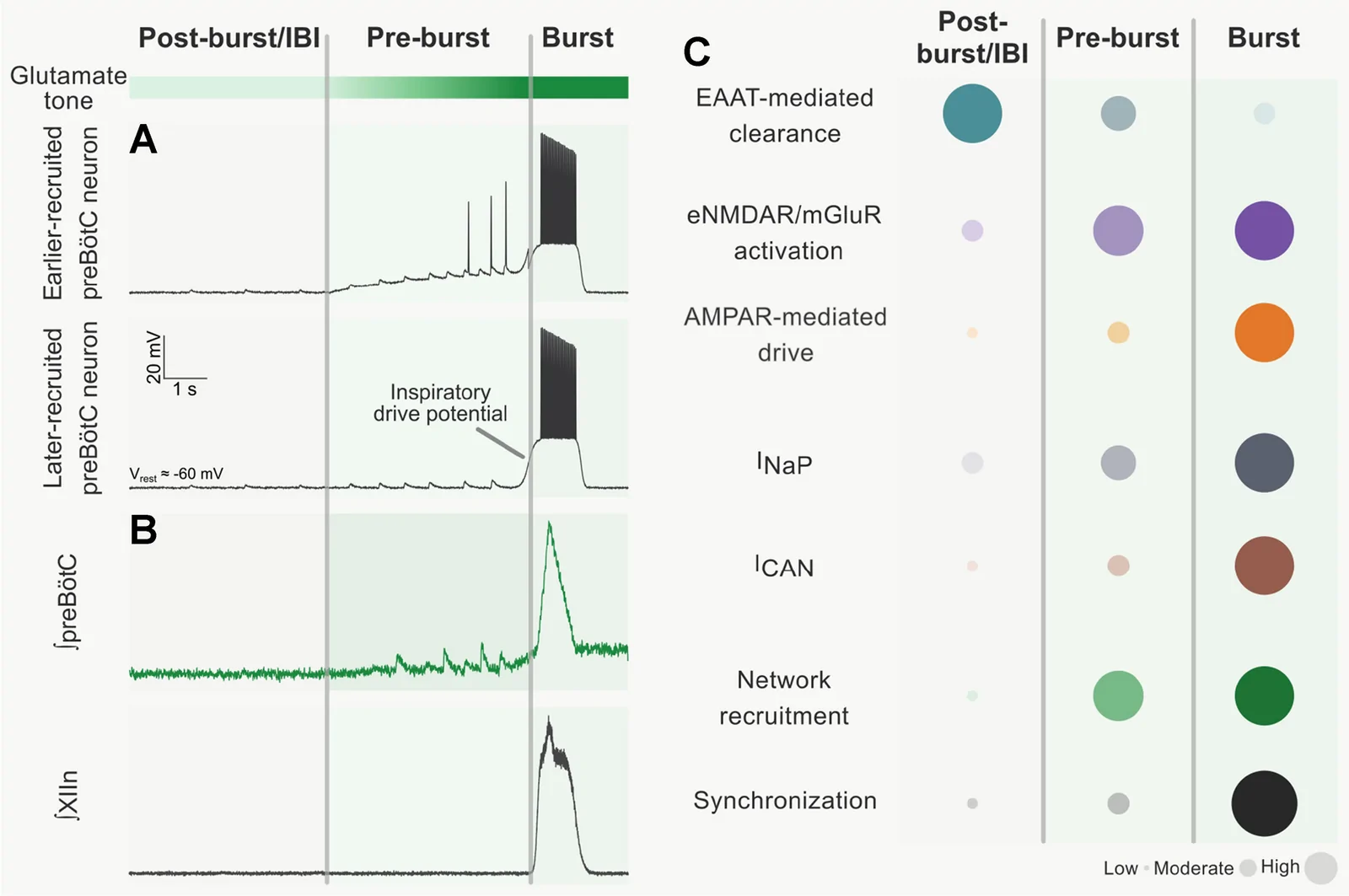
Predicted glutamate-tone dynamics during pre-burst threshold approach, recruitment, and synchronized inspiratory bursting. Working model illustrating how glutamate tone may contribute to a tonic excitability bias across the respiratory cycle. After an inspiratory burst, EAAT-mediated clearance is predicted to reduce glutamate tone toward a post-burst minimum. During the interburst interval, glutamate tone may recover as the balance shifts among residual spillover, sustained afferent input, non-synaptic sources, diffusion, and transporter-mediated clearance. This recovery is proposed to engage candidate glutamate-sensitive receptors, including eNMDARs and mGluRs, and bias subsets of preBötC neurons toward recruitment threshold. The traces illustrate earlier- and later-recruited preBötC neurons, not distinct neuronal classes. Once some neurons reach threshold, recurrent synaptic excitation amplifies recruitment and synchronization, generating the inspiratory drive potential, synchronized preBötC bursting, and hypoglossal (XIIn) motor output. The right panel summarizes predicted relative changes in clearance dominance, eNMDAR/mGluR activation, AMPAR-mediated drive, $I_{NaP}$, $I_{CAN}$, network recruitment, and synchronization across the post-burst, pre-burst, and burst phases. Dot size and shading represent schematic predictions of relative contribution or activation, not direct measurements. Abbreviations: **IBI**, interburst interval; **AMPAR**, AMPA receptor; **EAAT**, excitatory amino acid transporter; **eNMDAR**, peri- or extrasynaptic NMDA receptor; **mGluR**, metabotropic glutamate receptor; $\boldsymbol{I_{CAN}}$, Ca$^{2+}$-activated nonspecific cation current; $\boldsymbol{I_{NaP}}$, persistent Na$^{+}$ current; **sNMDAR**, synaptic NMDA receptor; $\boldsymbol{\int }$ **preBötC**, integrated preBötC population activity; $\boldsymbol{\int }$ **XIIn**, integrated hypoglossal motor output

The respiratory-cycle sequence predicted by this framework is illustrated in Fig. 2. After an inspiratory burst, EAAT-mediated clearance would be expected to reduce extracellular glutamate toward a post-burst minimum. During the interburst interval, glutamate tone may recover as the balance among residual spillover, sustained afferent input, non-synaptic sources, diffusion, and clearance shifts. If appropriately positioned glutamate-sensitive receptors detect this signal, such recovery could bias subsets of preBötC neurons toward recruitment threshold (Fig. 2C). This bias is proposed to be probabilistic rather than deterministic. Because preBötC neurons differ in intrinsic excitability, synaptic connectivity and effective synaptic weight, receptor distribution, and phase relationship to the burst, a modest ambient glutamate signal would be expected to affect most strongly those neurons already close to threshold.

Once a sufficient subset of neurons reaches threshold, synaptic glutamate release and recurrent excitation would dominate (Fig. 2A). Phasic synaptic drive would activate AMPARs and synaptic NMDARs, engage state-dependent intrinsic conductances such as $I_{NaP}$ and $I_{CAN}$, and generate the inspiratory drive potential and synchronized population burst that propagates to motor output [3] (Fig. 2B). Burst termination would reflect the combined action of glutamate clearance, receptor desensitization, intrinsic membrane mechanisms, network refractoriness, and inhibition. Thus, any effect of glutamate tone on recruitment or synchronization would be expressed within an excitatory-inhibitory microcircuit in which inhibitory neurons contribute to burst patterning, termination, and network stability. In this sequence, glutamate tone does not replace recurrent excitation or intrinsic conductances; it modifies the excitability landscape from which recruitment and synchronization emerge.

This framework also clarifies state dependence. Here, state dependence refers to any physiological or experimental condition that modifies extracellular glutamate availability, membrane potential, receptor activation, uptake, metabolism, or network excitability. Hypoxia, hypercapnia, altered metabolic demand, arousal, or neuromodulatory context could recalibrate preBötC excitability partly by shifting local glutamate tone. Commissural glutamatergic projections remain central for bilateral preBötC coupling; the present framework adds that local glutamate handling could tune how effectively this coupling is expressed on each side by modifying recruitment latency, probability, or robustness. The key prediction is not that glutamate tone alone generates rhythm, but that altering this extracellular variable should measurably change recruitment probability, recruitment timing, or rhythm robustness.

## Relationship to existing rhythm-generation models

The glutamate-tone framework is intended to complement, not replace, established models of preBötC rhythmogenesis. Recurrent glutamatergic excitation, intrinsic membrane conductances, and inhibitory circuitry remain central to neuronal recruitment, burst amplification, timing, patterning, and network stability [2–4]. Glutamate tone is therefore not proposed as a separate oscillator or as an alternative to AMPAR-mediated recurrent excitation, but as a candidate extracellular variable that may help set the excitability conditions under which established rhythm-generating mechanisms operate.

At the computational level, this framework offers one possible biological interpretation of part of $g_{\mathrm {tonic-e}}$, the tonic excitatory conductance used in Butera-Smith models to reproduce rhythmic bursting and state transitions [6]. We do not suggest that these models intended glutamate tone as the substrate of this parameter, nor that glutamate tone accounts for it entirely. Although phenomenologically useful, $g_{\mathrm {tonic-e}}$ is biologically composite: it may reflect intrinsic inward currents, leak conductances, neuromodulatory tone, extracellular ionic conditions, direct synaptic excitatory currents, and ambient extracellular glutamate.

These contributors are not fully independent. Synaptic glutamate release, including sustained afferent activity, may contribute to glutamate tone if released glutamate escapes the synaptic cleft, accumulates locally, or is incompletely cleared. The key distinction is therefore not the source of glutamate, but whether its functional effect is mediated by direct synaptic receptor activation or by ambient extracellular glutamate acting outside synaptic clefts. The value of the glutamate-tone hypothesis is that it identifies one component of baseline excitability that could, in principle, be measured, perturbed, and incorporated into future rhythm-generation models as experimentally constrained glutamate-release, clearance, and receptor-activation variables.

## From uncertainty to research program

The evidence reviewed here supports the biological plausibility of glutamate tone as a candidate contributor to preBötC excitability, but plausibility is not demonstration. No study has yet measured a compartment-specific ambient extracellular glutamate signal in the preBötC, shown that a defined receptor population detects such a signal under physiological conditions, or established a causal link between extracellular glutamate and recruitment timing, burst probability, synchronization, or rhythm robustness. The hypothesis therefore remains a biologically motivated framework for experimental testing, not an established mechanism.

**Fig. 3:**
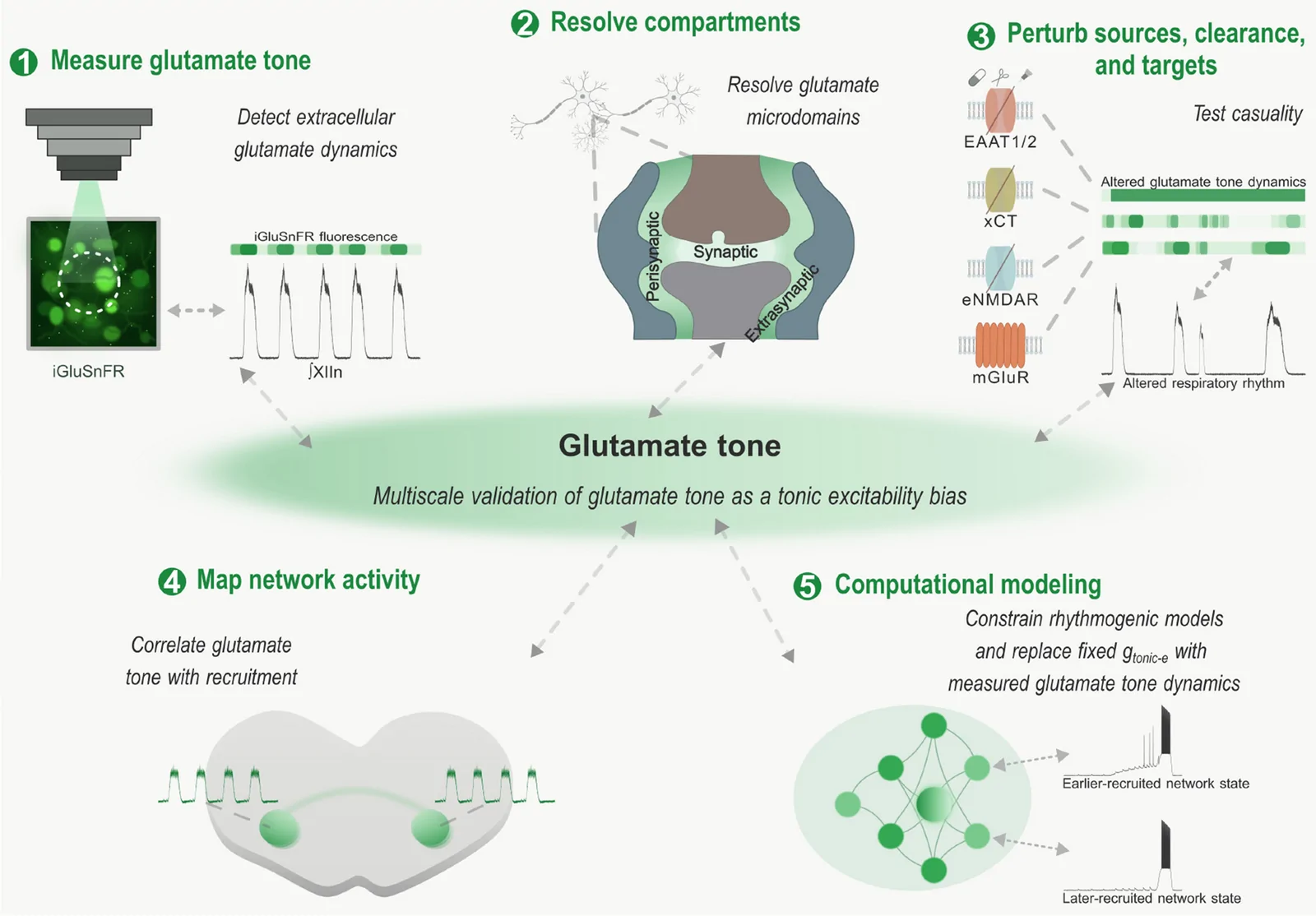
Experimental roadmap for testing glutamate tone as a rhythmogenesis-relevant excitability bias. Testing the glutamate-tone hypothesis requires linking measurement, perturbation, and modeling. Compartment-targeted glutamate sensors should define the timing and spatial distribution of extracellular glutamate signals across synaptic, perisynaptic, extrasynaptic, astrocyte-proximal, and neuropil domains. Targeted perturbation of EAAT1/2, xCT, eNMDARs, mGluRs, and AMPAR-mediated phasic transmission should determine whether glutamate-tone dynamics causally influence threshold approach, recruitment timing, burst probability, bilateral synchronization, or motor output. Computational models should then incorporate experimentally constrained glutamate release and clearance kinetics, transporter dynamics, receptor localization, co-agonist availability, Mg$^{2+}$ dependence, mGluR signaling, synaptic connectivity, and neuronal heterogeneity to replace, in part, fixed phenomenological $g_{\mathrm {tonic-e}}$ parameters. Abbreviations: **EAAT1/2**, excitatory amino acid transporters 1 and 2; **eNMDAR**, peri- or extrasynaptic NMDA receptor; **mGluR**, metabotropic glutamate receptor; **iGluSnFR**, intensity-based glutamate-sensing fluorescent reporter; **preBötC**, preBötzinger complex; **xCT**, cystine–glutamate exchanger; $\boldsymbol{\int ~XIIn}$, integrated hypoglossal motor output

A decisive test requires distinguishing receptor-mediated effects of ambient extracellular glutamate from direct synaptic receptor activation. This will require simultaneous measurement of extracellular glutamate, neuronal recruitment, and motor output, ideally with compartment-targeted glutamate sensors that resolve synaptic, perisynaptic, extrasynaptic, astrocyte-proximal, and neuropil domains (Fig. 3). Timing is central: a signal detected only after burst onset would support the interpretation that extracellular glutamate primarily reflects recurrent synaptic activation, whereas a signal that rises during the pre-burst interval, or covaries with recruitment probability before burst onset, would provide stronger support for a candidate excitability bias.

The receptor-identity and causality problems should be addressed together. For NMDARs, adult-stage subunit mapping should determine whether GluN2A/B/C/D heterogeneity reported in neonatal tissue persists in mature preBötC neurons and whether receptor populations with reduced Mg$^{2+}$ sensitivity are positioned to detect ambient glutamate. For mGluRs, subtype-specific localization and cell-type expression within the preBötC remain insufficiently defined. These studies should be paired with functional tests of tonic current, receptor kinetics, and receptor-specific effects on recruitment probability and burst timing. Manipulations of EAAT1/2, xCT, eNMDARs, mGluRs, and AMPAR-mediated phasic transmission should then determine whether altering extracellular glutamate availability or receptor sensitivity modifies the transition toward recruitment in a manner temporally consistent with an excitability bias (Fig. 3).

The local-versus-afferent contribution also needs to be resolved. Ambient extracellular glutamate in the preBötC may arise from local release-clearance balance, glutamatergic afferent input, or both. Comparing preparations that preserve major glutamatergic afferent pathways with preparations in which these inputs are selectively reduced would test whether local glutamate homeostasis and afferent drive contribute distinguishable components to the extracellular signal. State dependence should be tested under conditions that alter respiratory drive and network excitability, including hypoxia, hypercapnia, altered metabolic demand, arousal, and neuromodulatory context.

Most available evidence comes from neonatal *in vitro* preparations, in which slicing, continuous perfusion, elevated extracellular K$^{+}$, and reduced tissue architecture alter extracellular glutamate homeostasis. These preparations remain indispensable for mechanistic analysis, but they cannot by themselves establish that glutamate tone operates under physiological conditions. More intact preparations, and ultimately *in vivo* approaches combining glutamate sensors with neural and motor-output recordings, will be required.

The experiments also define observations that would weaken the framework. If extracellular glutamate changes consistently follow recurrent network activation, if compartment-targeted sensors fail to detect a pre-burst or slowly varying signal, or if perturbations of glutamate homeostasis alter burst amplitude without changing recruitment probability, burst timing, synchronization, or rhythm robustness, the hypothesis that glutamate tone biases preBötC excitability would be weakened. Conversely, evidence for a compartment-specific extracellular glutamate signal that precedes or covaries with recruitment, engages defined glutamate-sensitive receptors, and alters network excitability when selectively manipulated would support replacing part of the phenomenological $g_{\mathrm {tonic-e}}$ term with experimentally constrained glutamate-tone variables in future rhythm-generation models.

## Conclusion

Recurrent glutamatergic excitation, intrinsic membrane conductances, and inhibitory circuitry are well-established mechanisms for neuronal recruitment, synchronization, and inspiratory burst generation in the preBötC. What remains less clearly defined is whether extracellular processes help set the excitability state from which recruitment repeatedly emerges. We propose glutamate tone, defined here as ambient extracellular glutamate outside synaptic clefts, as one biologically plausible candidate for such an excitability-setting signal. In this framework, glutamate tone would not replace established rhythmogenic mechanisms, but could contribute to the conditions under which they operate.

The value of this framework is that it reframes part of a phenomenological excitability term as a set of experimentally testable biological variables: extracellular glutamate dynamics, clearance mechanisms, receptor localization, receptor biophysics, and state-dependent network recruitment. Current evidence does not demonstrate that glutamate tone regulates preBötC rhythmogenesis; rather, the framework defines the mechanisms and experimental criteria by which this possibility can be tested. Evidence for a compartment-specific extracellular glutamate signal that precedes or covaries with recruitment, engages defined glutamate-sensitive receptors, and alters recruitment timing, burst probability, synchronization, or rhythm robustness would support glutamate tone as a measurable component of preBötC excitability. Conversely, failure to meet these criteria would favor the interpretation that extracellular glutamate primarily reflects ongoing network activity rather than biasing recruitment or burst emergence.

## Data Availability

No datasets were generated or analysed during the current study.

## Declarations

### Clinical Trial Number

Not applicable. This manuscript is a mini-review and does not involve a clinical trial.
